# Outer Membrane Vesicles of Gram-Negative Bacteria: An Outlook on Biogenesis

**DOI:** 10.3389/fmicb.2021.557902

**Published:** 2021-03-04

**Authors:** Eric Daniel Avila-Calderón, María del Socorro Ruiz-Palma, Ma. Guadalupe Aguilera-Arreola, Norma Velázquez-Guadarrama, Enrico A. Ruiz, Zulema Gomez-Lunar, Sharon Witonsky, Araceli Contreras-Rodríguez

**Affiliations:** ^1^Departamento de Microbiología, Escuela Nacional de Ciencias Biológicas, Instituto Politécnico Nacional, México City, Mexico; ^2^Departamento de Biología Celular, Centro de Investigación y de Estudios Avanzados, Instituto Politécnico Nacional, CINVESTAV-IPN, México City, Mexico; ^3^División Químico Biológicas, Universidad Tecnológica de Tecámac, Tecámac, Mexico; ^4^Unidad de Investigación en enfermedades infecciosas, Hospital Infantil de México Federico Gómez, Ciudad de México, Mexico; ^5^Departamento de Zoología, Escuela Nacional de Ciencias Biológicas, Instituto Politécnico Nacional, México City, Mexico; ^6^Center for One Health Research, Virginia-Maryland College of Veterinary Medicine, Virginia Tech, Blacksburg, VA, United States; ^7^Large Animal Clinical Sciences, Virginia-Maryland College of Veterinary Medicine, Virginia Tech, Blacksburg, VA, United States

**Keywords:** outer membrane vesicles, bacterial vesicles, extracellular vesicles, OMVs biogenesis, phospholipids, LPS, PQS, flagellin

## Abstract

Outer membrane vesicles (OMVs) from Gram-negative bacteria were first described more than 50 years ago. However, the molecular mechanisms involved in biogenesis began to be studied only in the last few decades. Presently, the biogenesis and molecular mechanisms for their release are not completely known. This review covers the most recent information on cellular components involved in OMV biogenesis, such as lipoproteins and outer membrane proteins, lipopolysaccharide, phospholipids, quorum-sensing molecules, and flagella.

## Introduction

Outer membrane vesicles (OMVs) are nanostructures released by pathogenic and non-pathogenic Gram-negative bacteria *in vivo* and *in vitro*. OMVs range in size from 20 to 300 nm and are released during bacterial growth (Jan, 2017). These vesicles are formed from the bacterial outer membrane; thus, they contain phospholipids, lipopolysaccharide (LPS), outer membrane proteins (OMPs), and periplasmic proteins trapped during membrane budding (Jan, 2017). Because of their composition, these vesicles have been linked to many physiological processes, such as protein transport, nutrient acquisition, cell-intercellular communication, antibacterial activity, toxin delivery, and host-immune response modulation (Avila-Calderón et al., 2015).

Outer membrane vesicle generation begins with outer membrane bulging and ends with the release of vesicles into the external surroundings. The molecular mechanism for OMV production is still unclear. However, genetic and biochemical evidence has revealed some clues towards a better understanding of this complex process (Pathirana and Kaparakis-Liaskos, 2016).

The present review discusses new findings concerning OMV biogenesis as well as some important molecular determinants involved in vesicle formation, including lipoproteins, LPS, phospholipids, quorum-sensing molecules, and flagella.

### Models for OMV Biogenesis

The first observation of bacterial vesicles dates back to the 1960s, but only in recent decades has there been a significant increase in reports on the biogenesis, physiological roles, and applications of OMVs. Some of the first reports were performed using *Escherichia coli* strains, and researchers observed that the lysine-requiring mutant *E. coli* 12,408 produced “globules” surrounded by membranes with no evidence of cell lysis (Knox et al., 1966). Moreover, *E. coli* JC411 released vesicles containing lipids and proteins (Hoekstra et al., 1976). Other experiments using *E. coli* W3110 showed that membrane vesicles were released from the cell surface after heating the cells at 55°C. These vesicles contained lipid, LPS, and protein compositions similar to those of the outer membrane from whole cells (Katsui et al., 1982). After these findings were reported, the number of published studies concerning OMVs has increased (Gamazo and Moriyón, 1987; Grenier and Bélanger, 1991; Kadurugamuwa and Beveridge, 1995).

Three models propose a role for lipoproteins, LPS, and peptidoglycan in the biogenesis of OMVs. Recently, new reports contributed to the knowledge of OMV production, some of which agree with the early models proposed, whereas others shed light on new molecules involved in vesiculation. In this section, the three early models are described initially; in the subsequent section, new insights into the molecular determinants involved in OMVs are described.

The first model explains the formation of OMVs due to the low number of lipoproteins attached to the peptidoglycan layer. The low number of lipoprotein linkages leads to outer membrane bulging, affecting vesicle production. In 1976, Hoekstra et al. determined the number of lipoproteins found in the outer membrane of *E. coli* as well as those contained in the vesicles. These authors reported fewer lipoproteins in the vesicles than in the outer membrane. Based on these results, the authors proposed that vesicles are released from outer membrane sites with few lipoprotein linkages (Hoekstra et al., 1976; Mashburn-Warren and Whiteley, 2006). The second model was based on the presence of peptidoglycan residues with autolysins in the OMVs. This model proposes that during synthesis of the peptidoglycan layer, sites exist where the concentration of peptidoglycan is higher, causing protrusions in the outer membrane and indicating the beginning of vesicle formation. An important finding that supported this model was the presence of muramic acid, a known peptidoglycan layer precursor, in OMVs purified from *Porphyromonas gingivalis* (Zhou et al., 1998). Furthermore, mutation of an autolysin involved in peptidoglycan replacement increased the synthesis of OMVs (Hayashi et al., 2002). These findings suggest that the accumulation of peptidoglycan residues increases outer membrane bulging, triggering the release of OMVs (Zhou et al., 1998; Hayashi et al., 2002). The third and last model involves the electric charge of LPS in OMV formation. *Pseudomonas aeruginosa* produces two types of LPS: negatively charged LPS and neutrally charged LPS. OMVs released by *P. aeruginosa* cultured under oxidative stress conditions primarily comprise negatively charged LPS. Therefore, an increase in negatively charged LPS within the cell envelope was proposed to favor the release of OMVs because of the repulsion caused by negative charges in the outer membrane (Kadurugamuwa and Beveridge, 1995; Sabra et al., 2003).

These models highlighted the importance of lipoproteins, LPS, and peptidoglycan during OMV formation. However, it remains unknown whether these mechanisms act in tandem. Some of the most important and widely accepted mechanisms for OMV formation are outlined in the following sections.

### Some Lipoproteins and the Outer Membrane Protein OmpA are Involved in OMV Biogenesis

The envelope of Gram-negative bacteria is made up of the inner and outer membranes, which are separated by the periplasmic space. Peptidoglycan is bound to proteins in the outer and inner membranes through covalent and noncovalent bonds (Silhavy et al., 2010). Peptidoglycan is composed of linear glycans (g β-1,4-connected N-acetylglucosamine and N-acetylmuramic acid) that are cross-linked by short peptides (Pazos and Peters, 2019). Braun’s lipoprotein, commonly referred to as Lpp, is the major lipoprotein in *E. coli* and is the only lipoprotein covalently that is linked to the peptidoglycan layer and plays a unique role in the envelope architecture. The N-terminal domain of Lpp is acylated and inserted into to the outer membrane, while the C-terminal domain is covalently linked to the peptidoglycan layer (Lee and Inouye, 1974). OmpA in *E. coli* is a β-barrel, and its C-terminal domain interacts with peptidoglycan through a 20-aa residue linker region (Smith et al., 2007). In 2017, Samsudin et al. have shown that interactions among OmpA, peptidoglycan, and Lpp are essential in maintaining the integrity of the cellular envelopes. The research team showed that Lpp aids in the interaction of monomeric OmpA with the peptidoglycan layer. In the absence of Lpp, the C-terminal domain of OmpA binds to the outer membrane. Additionally, an OmpA homodimer can easily interact with the peptidoglycan layer in the absence of Lpp (Samsudin et al., 2017). These lipoproteins and outer membrane proteins are associated with outer membrane stability.

Initially, the production of “blebs” (OMVs) was considered an alteration or instability of the outer membrane rather than a physiological phenomenon. For example, in 1969, Koike et al. observed bleb formation on the surface of *E. coli* after treatment with polymyxin B. However, the same authors observed that polymyxin B induced outer membrane projections rather than blebs in *P. aeruginosa*. These projections diminished as the concentration of the antibiotic was decreased. Thus, blebs and outer membrane projections were considered to be affected by the action of the antibiotic on the outer membrane (Koike et al., 1969). Subsequently, the release of OMVs was observed in more bacterial species, and vesicles were found not to be the product of cell lysis (Zhou et al., 1998). The exclusion of some periplasmic and outer membrane components and enrichment of other components in vesicles led to the consideration of the existence of a specific mechanism to select molecules carried on OMVs (Kadurugamuwa and Beveridge, 1995; Horstman and Kuehn 2000; Kato et al., 2002; Bonnington and Kuehn, 2014). Based on this background, “OMV release” is the product of a physiological phenomenon but not the product of cell lysis or membrane instability.

**Figure 1:**
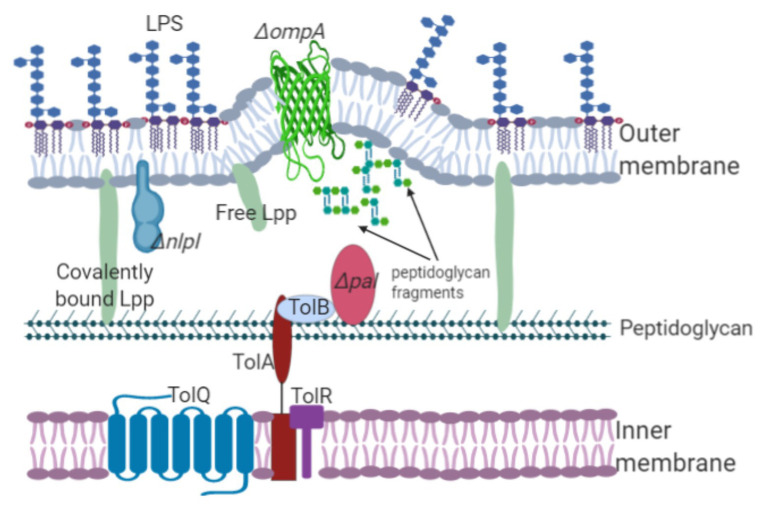
Lipoproteins and outer membrane proteins are involved in OMV biogenesis. The Lpp, NlpI, OmpA, and Tol-Pal members maintain the stability of cellular envelopes joining the peptidoglycan layer with the inner membrane. Interruption or deletion of the genes encoding these proteins decrease the number of linkages, inducing OMV formation. For example, mutation of the *pal* gene decreases linkage with the outer membrane, leading to membrane protrusion and OMV release. Additionally, the accumulation of components in the periplasm, such as peptidoglycan precursors, triggers vesicle formation.

In 1976 and 1978, the first molecular evidence regarding OMV biogenesis was revealed by Weigand et al. whose results were obtained using *Salmonella enterica* serovar Typhimurium (hereafter referred to as *Salmonella typhimurium*) and Suzuki et al. whose work focused on *E. coli*. Their works showed that the *E. coli lpo* mutant and *S. typhimurium lkyD* mutant lacking the murein lipoprotein (the product of these genes was later designated Braun’s lipoprotein or Lpp) released “blebs” from the outer membrane. Although Suzuki et al. (1978) observed the production of blebs from *Salmonella* cells, Weigand et al. (1976) reported bleb production at the septal region in *E. coli*. Bernadac et al. (1998) studied two mutated *E. coli* strains, the *lpp* and *ompA* mutants, both of which exhibited a hypervesiculation phenotype. Similarly, Lpp was also associated with OMV production in bacterial species such as *Yersinia pestis* (Eddy et al., 2014). The hypervesiculation phenotype was also observed in *Acinetobacter baumannii* and *Vibrio cholerae ompA* (Moon et al., 2012; Valeru et al., 2014). *A. baumannii*, *V. cholerae*, and other Gram-negative bacteria with a mutation in the *omp*A gene could not establish bonds between the peptidoglycan and the outer membrane, leading to OMV overproduction (Figure 1; Jin et al., 2011; Moon et al., 2012). These results demonstrate that lipoproteins and the outer membrane proteins involved in membrane stability are linked to OMV biogenesis.

The role of Lpp and OmpA in the formation of OMVs has also been analyzed. However, some discrepancies were observed in the results obtained by different research groups. In 2009, Deatherage et al. analyzed the effect on OMV formation in *S. typhimurium* LT2 caused by specific mutations in the *lpp* and *omp*A genes. Further experiments on the *S. typhimurium* LT2 *tolA*, *tolB*, and *pal* mutants suggested that they could not form outer-membrane-peptidoglycan-inner membrane linkages, and vesicle formation was observed around the cell body. Weigand et al. (1976) reported OMV formation in the septa of the *S. typhimurium lpp* mutant. However, Deatherage et al. (2009) observed vesicle formation in the cell bodies of different *S. typhimurium lpp* gene mutants. Differences observed in both studies can be attributed to *S. typhimurium* harboring two copies of the lipoprotein gene that are separated by 82 bp (*lppA* and *lppB*). Deatherage et al. (2009) mutated both *lpp* genes, while Weigand mutated only one of them. These findings showed two possible hypotheses: (i) the bonds between the outer membrane and peptidoglycan layer dissociate, leading to OMV release into the periphery of the cell body (Deatherage et al., 2009); and (ii) during bacterial division, the bonds between the peptidoglycan layer and inner membrane decrease within the division septum, affecting the number of linkages among the cytoplasmic membrane, cell wall, and outer membrane, and leading to membrane protrusion and vesicle release (Deatherage et al., 2009). OMVs are released from either the septa or cell body likely because of specific interactions among Lpp, OmpA, and peptidoglycan. OmpA, as a monomer or homodimer, interacts with peptidoglycan and the outer membrane in the presence or absence of Lpp. Most likely, the presence of OmpA as a monomer or homodimer and its distribution on the outer membrane determine the sites where vesicles can form.

Deatherage et al. (2009) and Nevermann et al. (2019) treated *S. typhi* and *S. typhimurium* LT2 *ompA* mutants with deoxycholate together with vancomycin and evaluated bacterial viability. Nevermann et al. (2019) reported that the *S. typhi ompA* mutant was sensitive to deoxycholate and that the *S. typhimurium* ATCC14028s *ompA* mutant was sensitive to vancomycin. However, Deatherage et al. (2009) found that the *S. typhimurium* LT2 *ompA* mutant was not sensitive to deoxycholate. The sensitivity of the *S. typhi* and *S. typhimurium ompA* mutants to deoxycholate suggests defective outer membrane stability. Additionally, the hypervesiculating phenotype might be expressed because of defective outer membrane permeability. Although *S. typhi* and *S. typhimurium* are phylogenetically closely related, differences observed in the membrane stability of the mutants in the study suggest a different role for the OmpA lipoprotein in OMV biogenesis.

The accumulation of periplasmic compounds, including proteins and peptidoglycan, induces membrane protrusion and vesicle release (Hayashi et al., 2002; McBroom and Kuehn, 2007). Schwechheimer et al. (2014) analyzed the relationship among the accumulation of periplasmic compounds, Lpp linkages, and OMV release. The effects of the periplasmic content on OMV formation were analyzed using three *E. coli* mutants: (i) *E. coli ΔampGΔamiD*, a mutant defective in the transport of peptidoglycan-recycling residues; (ii) *E. coli ΔrfaC*, *ΔrfaG*, and *ΔrfaP* mutants defective in the transport and assembly of LPS; (iii) *E. coli* Δ*degP*, a mutant that cannot degrade misfolded peptides in the periplasm. The hypervesiculation phenotype was exhibited by the three *E. coli* mutants likely because of the accumulation of peptidoglycan fragments, LPS, and proteins in the periplasm. However, the overall number of Lpp bonds were not affected because the levels of Lpp cross-linking were similar to those observed in the wild-type strain (Schwechheimer et al., 2014). By contrast, the *E. coli ΔmepAΔdacBΔpbpG* mutant, defective in three peptidoglycan hydrolases, showed a decreased number of Lpp-peptidoglycan linkages and increased OMV production (Schwechheimer et al., 2014). The vesiculation process was analyzed for *E. coli* strains displaying deficient genes encoding L,D-transpeptidases (*ynhG* and *ycbB*). *ynhG* (now renamed *ldtE*) and *ycbB* (*ldtD*) encode enzymes that catalyze the formation of meso-diaminopimelyl → meso-diaminopimelyl crosslinks (also called DAP→DAP or 3–3 cross-links) in peptidoglycan (Magnet et al., 2007). The *E. coli ΔynhGΔycbB* mutant produced fewer OMVs than the wild-type strain, and the number of Lpp cross-links in this mutant increased (Schwechheimer et al., 2014). Therefore, the authors propose that the *E. coli ΔynhGΔycbB* mutant, lacking DAP-DAP linkages, exhibits Lpp and peptidoglycan cross-links formed randomly around the cell body, decreasing OMV release. These data revealed that the modulation of the peptidoglycan structure leads to a decrease in Lpp linkages and an increase in OMV production, while a decrease in OMV production is related to an increase in covalently bound Lpp to peptidoglycan (Schwechheimer et al., 2014).

Another lipoprotein found to be involved in OMV biogenesis is NlpA, which is an inner membrane lipoprotein. Mutation of this protein decreases OMV production in *E. coli* (Yamaguchi and Inouye, 1988; McBroom et al., 2006; Schwechheimer and Kuehn, 2013). Furthermore, Schwechheimer et al. (2014) observed that the single mutation of the inner-membrane-anchored lipoprotein NlpA decreased vesiculation in *E. coli*, while a specific mutation of the *ompA* gene increased OMV production. The *E. coli ΔnlpAΔompA* double mutant exhibited heightened vesiculation (Schwechheimer et al., 2014). However, the *E. coli ΔycfSΔybiSΔerfKΔnlpA* mutant (defective in L,D-transpeptidases involved in the covalent crosslink between Lpp and peptidoglycan, and NlpA) showed increased vesiculation compared with that of the *E. coli ΔycfSΔybiSΔerfK* mutant. The heightened vesiculation observed in both the *E. coli ΔnlpAΔompA* and *E. coli ΔycfΔybiΔerfKΔnlpA* mutants can be explained in terms of NlpA, which provides stabilization of sites of the bacterial envelope necessary for Lpp and OmpA linkages (Schwechheimer et al., 2014). In 1998, Bernadac et al. used a Tol-Pal system mutated *E. coli* strain to purify and analyze OMVs using electron microscopy. The research team observed that the *tolA*, *tolQ*, and *tolR* mutants displayed a hypervesiculation phenotype, whereas the *tolB* and *pal* mutants showed a reduced vesicle production (Bernadac et al., 1998). The Tol-Pal system comprises proteins associated with outer membrane integrity. TolA, TolQ, and TolR link the inner membrane to peptidoglycan, whereas TolB and the peptidoglycan-associated lipoprotein (Pal) interact with the outer membrane (Gerding et al., 2007). McBroom et al. (2006) phenotyped random transposon mutants and found that the *E. coli tolA*, *tolB*, and *pal* mutants showed a hypervesiculation phenotype. Similar results of hypervesiculation were observed in the *P. aeruginosa oprL* and *oprI* mutants (Wessel et al., 2013). OprL and OprI are homologs to the Pal and Lpp proteins, respectively (Hancock et al., 1990). *P. aeruginosa oprI* and *oprF* mutants produced more OMVs than wild-type *P. aeruginosa* (Wessel et al., 2013). A recent study by Nevermann et al. (2019) identified genes related to OMV biogenesis in *S. typhi* employing transposon mutagenesis. Mutation of the *tolR* gene led to the expression of the hypervesiculation phenotype. The *S. typhi tolR* mutant was treated with deoxycholate together with vancomycin. Subsequently, cell viability was measured to evaluate whether membrane integrity had been affected. Following treatment with deoxycholate, neither cell growth nor hypervesiculation in the *S. typhi tolR* mutants was affected. These results indicated that OMV production was due to defective lipoprotein linkages. Tol-Pal members are highly homologous (possibly because of speciation events, gene duplication, and/or lipoprotein redundancy), and their distinct functions may be essential to OMV biogenesis.

NlpI is an outer-membrane-anchored lipoprotein whose function remains unknown. Although it was previously associated with cell division in *E. coli*, it has also been related to biofilm formation in *S. typhimurium* (Ohara et al., 1999). Inactivation of the *nlpI* gene in *E. coli* resulted in abnormal cell division and formation of membrane projections, whereas overexpression of this gene inhibited cell growth (Rouf et al., 2011; Banzhaf et al., 2020). The *S. typhimurium* ATCC14028s *nlpI* mutant expressed the hypervesiculation phenotype and was more resistant to vancomycin than the *S. typhi nlpI* mutant (Nevermann et al., 2019). However, the *E. coli nlpI* mutant displayed the hypervesiculation phenotype (McBroom et al., 2006). Schwechheimer et al. (2015) obtained an *E. coli nlpI* mutant displaying the hypervesiculation phenotype but no sensitivity to deoxycholate, concluding that the hypervesiculation phenotype observed was neither due to defects in membrane integrity nor cell lysis. NlpI regulates the activity of the peptidoglycan hydrolases Spr and PBP4, which play an essential role in cell wall renewal. Mutation of the *nlp*I gene in *E. coli* increased Spr and PBP-4 activity, as well as peptidoglycan cleavage and peptidoglycan synthesis. The increased activity of Spr and PBP-4 hydrolyses observed in the *E. coli nlpI* mutant led to decreased linkages between Lpp and peptidoglycan and vesicle overproduction. Most likely, peptidoglycan dynamics (i.e., growth and renewal) are regulated by NlpI in the *E. coli* wild-type strain. However, peptidoglycan hydrolases Spr and PBP-4 decrease Lpp-peptidoglycan linkages, inducing outer membrane protrusion and subsequent OMV formation. Importantly, peptidoglycan dynamics and/or Lpp-peptidoglycan linkages are also affected by different hydrolases that are not regulated by NlpI. Based on these findings, NlpI lipoprotein is likely closely associated with OMV formation (Schwechheimer et al., 2015).

### Bacterial LPS Plays an Essential Role in OMV Production

The outer membrane is an asymmetric structure comprising an inner leaflet made up of phospholipids along with proteins and an outer leaflet that contains phospholipids, proteins, and LPS. LPS is an essential structure for Gram-negative bacteria and is the most abundant antigen found on their cell surface; for example, the outer membrane of *E. coli* and *Salmonella* genera can contain up to 75% LPS (Klein and Raina, 2019).

The structure of smooth LPS comprises an O-side chain, an intermediate region known as the core oligosaccharide and a glycolipid (lipid A) anchored to the outer membrane. Although smooth strains contain the O-side chain in their LPS, rough strains do not (Raetz and Whitfield, 2002). The core oligosaccharide is divided into two sections: the inner core, proximal to lipid A, and the outer core, which is the attachment site for the O-antigen. Lipid A can become chemically modified. For example, phosphoethanolamine, 4-amino-4-deoxy-L-arabinose (L-Ara4N), and additional palmitate groups were added (Raetz and Whitfield, 2002). Such lipid A modifications make the bacteria more resistant to cationic antibacterial peptides and polymyxin (Raetz and Whitfield, 2002).

*P. aeruginosa* produces two variants of the O-side chain antigen: the common polysaccharide antigen, also called the CPA or A-band (a short, neutrally charged molecule), together with the O-specific antigen, also referred to as the OSA or B-band (a highly immunogenic, negatively charged molecule; Lam et al., 2011). Both OSA and CPA were detected in OMVs obtained from a *P. aeruginosa* strain cultured in the presence and absence of gentamicin. OMVs exhibited a higher concentration of OSA in the absence of gentamicin; however, in the presence of this antibiotic, CPA was hardly detected within the vesicles (Kadurugamuwa and Beveridge, 1995). The addition of gentamicin (a polycation) to *P. aeruginosa* cultures modified electric charges in the outer membrane, affecting LPS packing into the OMVs. The role of negatively charged LPS in the biogenesis of OMVs was confirmed using *P. aeruginosa* CPA^−^, OSAB^−^, and CPA^−^OSA^−^ mutants. In 2003, Nguyen et al. confirmed that the OSA^−^LPS complex also contributes to OMV formation because the *P. aeruginosa* CPA^−^ mutant, which only synthesizes negatively charged LPS, released larger OMVs than those released by the OSA^−^ mutant (Nguyen et al., 2003). These data showing that OMVs contain a higher concentration of negatively charged LPS suggest that OSA plays a role in vesicle formation.

In 2015, Cahill et al. while working with the *Klebsiella pneumoniae wbbO* mutant, a glycosyltransferase-defective strain lacking the O-side chain, reported that the release of OMVs was not affected, whereas, the outer membrane and vesicles from the *K. pneumoniae wbbO* mutant exhibited a different protein profile and were quite distinct from the vesicles of the wild-type strain. Furthermore, vesicles released by the *K. pneumoniae wbbO* mutant contained a higher concentration of proteins associated with posttranslational modification, protein turnover, and chaperones. However, OMVs produced by the *K. pneumoniae* wild-type strain contained proteins involved in cell wall, membrane, and envelope biosynthesis (Cahill et al., 2015). *P. gingivalis*, an etiologic agent of chronic periodontitis, also expresses two types of LPS, neutrally charged LPS (O-LPS) and negatively charged LPS (A-LPS; Paramonov et al., 2005, 2009). Studies performed on *P. gingivalis porS* and *waaL P* mutants demonstrated that neither A-LPS nor O-LPS is essential for OMV biogenesis. The *P. gingivalis porS* mutant, lacking the flippase PorS, did not display the O-antigen in lipid A. However, the *P. gingivalis waaL* mutant lacks the O-antigen ligase WaaL, generating rough cells. Nonetheless, the electrophoretic profile of proteins associated with OMVs produced by the *P. gingivalis waaL* mutant was different from that obtained from the wild-type strain (Haurat et al., 2011). These results suggest that negatively charged LPS influence protein packing into OMVs.

In 2014, Murphy et al. analyzed lipid concentrations in OMVs released from *P. aeruginosa* CPA^−^, OSA^−^, and CPA^−^OSA^−^ mutants. No differences were found in either the concentration of lipids or number of vesicles released by all three mutants (Murphy et al., 2014). Most likely, repulsion between the core and lipid A produced an inducing effect on the *P. aeruginosa* CPA^−^OSA^−^ double mutant but not on the CPA or OSA single mutant. By contrast, the double mutant exhibited an increased number of OMVs, but no change in size was observed. OMVs from the *P. aeruginosa* CPA^−^OSA^−^ mutant and wild-type strain contain proteins sharing similar functions. Nevertheless, OMVs from the *P. aeruginosa* CPA^−^ mutant showed the highest accumulation of proteins involved in the transport of small molecules. The *P. aeruginosa* OSA^−^ mutant displayed the highest proportion of proteins involved in adaptation, protection, and transcriptional regulation (Murphy et al., 2014). A high content of periplasmic proteins and a low number of OMPs were detected in OMVs from the *P. aeruginosa* OSA^−^ mutant. Conversely, high numbers of OMPs and periplasmic proteins were observed in OMVs from the *P. aeruginosa* CPA^−^OSA^−^ double mutant, CPA^−^ mutant and wild-type strain (Murphy et al., 2014). These results suggest that OSA-LPS plays an important role in the selection of proteins. Proteins interacting with specific LPS lead to OMVs with different contents due to LPS composition. It is highly probable that the lack of the O-side chain modifies the electric charge on the surface of the membrane, impairing electrostatic interactions between proteins and electrically charged LPS, and ultimately affecting the protein packing of OMVs.

**Figure 2:**
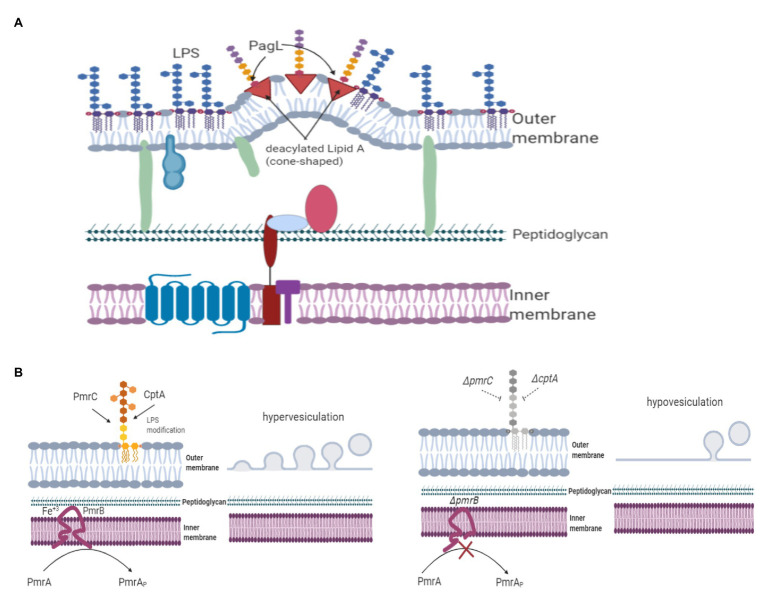
LPS modifications are implicated in OMV release. Negatively charged LPS causes an outer membrane imbalance, subsequent membrane protrusion, and OMV release. LPS modifications also contribute to OMV formation. **(A)** The *S. typhimurium* strain expressing PagL without the PhoPQ control produces more OMVs than the wild-type strain. The outer membrane of *S. typhimurium* expressing PagL shows that hexa-acylated lipid A is predominant, while OMVs primarily contain penta-acylated lipid A. Thus, either the cylindrical or inverted-cone shape decreases hydrophobic interactions, leading to membrane protrusion and favoring OMV formation. **(B)** *Citrobacter rodentium* uses a two-component system to modify LPS. PmrB, a member of this system, senses high Fe^+3^ concentrations and regulates the modification of lipid A. *C. rodentium ΔpmrB* cannot add groups to lipid A, leading to increased vesiculation.

PagL is an enzyme that modifies lipid A by removing the acyl chain at the 3-position of the disaccharide backbone (King et al., 2009). In *S. typhimurium*, a deacylated lipid A (i.e., penta-acylated lipid A) modified by PagL makes LPS less detectable to Toll-like receptor 4 of the mouse B-cell line (Kawasaki et al., 2004). PagL activity is regulated by a two-component system (represented as PhoP/PhoQ), Mg^+2^ and temperature, being less active at low Mg^+2^ concentrations and temperatures (Trent et al., 2001a; Ernst et al., 2006). OMVs from *P. gingivalis* displayed higher concentrations of deacylated lipids (Haurat et al., 2011). Therefore, the effect of lipid A deacylation on OMV biogenesis was evaluated using the expression of the PagL enzyme. The *pagL* gene was cloned using a low-copy expression vector lacking a control from the PhoP/PhoQ two-component system. *S. typhimurium* expressing PagL produced almost four times more OMVs than the wild-type strain (Elhenawy et al., 2016). The outer membrane of the *S. typhimurium* strain expressing PagL showed that hexa-acylated lipid A was predominant, while OMVs mainly contained penta-acylated lipid A (Elhenawy et al., 2016). The authors explained that these differences are likely based on conformational changes in the lipid A structure because hexa-acylated lipid A has a conical shape, while deacylated lipid A has a cylindrical or inverted cone shape. Thus, either the cylindrical or inverted-cone shape decreases hydrophobic interactions, leading to membrane protrusion and favoring OMV formation (Figure 2A; Schromm et al., 2000; Elhenawy et al., 2016). The presence of deacylated lipid A in other Gram-negative bacteria should be investigated to determine whether this is a common molecule implicated in OMV biogenesis. The quantitative comparison analysis of subtypes of LPS in OMVs and outer membranes of wild type *S. typhimurium* undergoing environmental shift conditions that do not rely on PagL supported an additional role for polymorphic regulation of membrane LPS composition in vesiculation (Bonnington and Kuehn, 2016). Nonlamellar types of lipids, which have an overall conical shape and a preference for the hexagonal phase, are thought to aid in the formation of nonbilayer structures. Accordingly, both the geometry and the propensity of individual membrane components to form nonbilayer lipid phases in the membrane are both likely to be important to vesiculation processes.

*Citrobacter rodentium*, a murine enteric pathogen, lacks the *pagL* gene. This bacterium uses two-component systems to modify LPS, and these systems may indirectly regulate OMV production, similar to PagL. One of these systems is PmrAB, a two-component system regulated by low pH and Fe^+3^ concentration. PmrAB regulates the expression of the *pmrD* gene. PmrB senses high Fe^+3^ concentrations. Once PmrA is activated through phosphorylation, it triggers the differential expression of *pmrC* (also known as *eptA*) and *cptA*; both *pmrC* and *cptA* catalyze LPS modifications (Perez and Groisman, 2007). In *Salmonella enterica*, PmrC and CptA link phosphoethanolamine, while ArnT links 4-amino-4-deoxy-L arabinose to lipid A and the LPS core; these bonds are regulated by PmrAB (Trent et al., 2001b; Lee et al., 2004; Tamayo et al., 2005). Once 4-amino-4-deoxy-L arabinose and phosphoethanolamine are linked to lipid A and the core, the synthesis of negatively charged LPS is decreased, and molecules attached to LPS enhance resistance to antimicrobial peptides and oxidative stress (Sinha et al., 2019). The *C. rodentium ΔcptA*, *ΔpmrC*, and *ΔpmrAB* mutants produce more OMVs than the wild-type strain (Sinha et al., 2019). Furthermore, decreased vesiculation was observed in *C. rodentium* strains overexpressing the *pmrC* (*eptA*) or *cptA* genes, as shown in Figure 2B (Sinha et al., 2019). PmrC and CptA are putative transferases in *C. rodentium*; hence, mutations of these genes could lead to decreased modification of LPS with phosphoethanolamine, increasing the overall negative charge of LPS and causing outer-membrane curvature imbalance, subsequent membrane protrusion, and OMV release.

Nevermann et al. (2019) demonstrated that the *waaC* and *rfaE* genes are also associated with OMV biogenesis in *S. typhi*. Additionally, RfaE has been associated with heptose (Hep) precursors necessary for inner-core LPS assembly in *E. coli* and *S. typhimurium* (Valvano et al., 2002). Heptoses are found in the oligosaccharide core, and they play a fundamental role in outer membrane stability and the crosslinking of adjacent LPS; they also interact with the positive charges of certain proteins (Heinrichs et al., 1998). Heptosyltransferase I (HepI or WaaC) catalyzes the addition of the first heptose to the inner Kdo (3-deoxy-D-manno-oct-2-ulosonic acid; Czyzyk et al., 2011). The *S. typhi waaC* mutant produced fewer vesicles than the wild-type strain, while the OMV protein profiles from both the mutant and wild-type strains were similar. Additionally, the *S. typhi rfaE* mutant produced more vesicles; however, when comparing OMV protein profiles from both the mutant and wild-type strains, relevant differences could clearly be observed. Lateral interactions with LPS molecules provide a significant fraction of the driving force necessary to establish lipid asymmetry in the outer membrane (Nikaido, 2003). Regarding the *S. typhi rfaE* mutant, it is likely that a defective link of heptose in the inner core alters its assembly, modifying certain interactions between positively and negatively charged LPS groups, and affecting the overall protein interaction with LPS. This finding could explain why the protein composition of OMVs from the *S. typhi rfaE* mutant was different from that obtained for OMVs from the wild-type strain. WaaC and RfaE induced an opposite effect on vesiculation. Mutation of the *waaC* gene led to the expression of the hypovesiculation phenotype, while mutation of the *rfaE* gene induced a hypervesiculation phenotype. These data confirmed the same effects on the OMV phenotypes by *waaC* and *rfaE* mutants found for *E. coli* (Kulp et al., 2015). Further research should focus on these enzymes, which are associated with inner core assembly, and how they contribute to OMV formation.

### Contribution of Phospholipids to OMV Biogenesis

Most reports concerning OMV biogenesis focus on the role of lipoproteins and LPS. However, little is known about phospholipids, which are the major components of OMVs. The main phospholipids comprising the outer membrane of *E. coli*, in order of proportion, are phosphatidylethanolamine (PE), phosphatidylglycerol (PG), and cardiolipin (CL; Raetz and Dowhan, 1990). Using thin-layer chromatography, Horstman and Kuehn (2000) determined that the phospholipid composition of the outer membranes of the *E. coli* HB101 and ETEC 2 strains, as well as their respective vesicles, share similar components, including PE, PG, and CL. OMVs from *Actinobacillus actinomycetemcomitans* displayed higher CL and PE concentrations and a low proportion of unidentified lipids, which are not present in the outer membrane (Kato et al., 2002). OMVs from *P. aeruginosa* showed high PG and stearic acid concentrations, but a low percentage of unsaturated fatty acids released from vesicles composed of a rigid membrane (Tashiro et al., 2011). The *orf5* and *plsC1* genes of *Shewanella livingstonesis* play an essential role in eicosapentaenoic acid biosynthesis, while *plsC1* and *plsC4* code for acyltransferases that are involved in the synthesis of membrane phospholipids. Mutation of *orf5*, *plsC1*, and *plsC4* in *S. livingstonesis* increased the production of vesicles (Yokoyama et al., 2017).

Gram-negative bacteria maintain asymmetric distribution of phospholipids in their membranes through the translocation of phospholipids from the inner membrane to the inner leaflet of the outer membrane (known as phospholipid anterograde transport) and inversely (known as phospholipid retrograde transport; Shrivastava and Chng, 2019). Regarding anterograde transport, the molecular determinants implicated in the process are still not fully understood; however, the PbgA/YejM lipoprotein is involved in CL transport from the inner to the outer membrane in *Shigella flexneri* and *S. typhimurium* (Dalebroux et al., 2015; Rossi et al., 2017). Regarding retrograde transport, two systems have been described: (i) the Tol-Pal system is involved in the movement of phospholipids from the inner leaflet of the outer membrane to the inner membrane; (ii) the OmpC-Mla system is responsible for outer membrane asymmetry by transporting misplaced phospholipids found in the outer leaflet of the outer membrane to the inner membrane (Chong et al., 2015; Shrivastava et al., 2017). The MlaA lipoprotein interacts with OmpC, which is embedded in the outer membrane and removes phospholipids in the outer leaflet of the outer membrane to another component of the system the MlaC protein. Subsequently, MlaC delivers these phospholipids to the MlaFEDB complex located in the inner membrane. This complex can then reintegrate these phospholipids to the inner membrane (Malinverni and Silhavy, 2009; Chong et al., 2015). Mutation of *mla* in *E. coli* induces vesicle production at the septa, increases hepta-acylated LPS, and promotes cell death (Sutterlin et al., 2016). Vesicle overproduction decreases the lipid levels in the outer membrane that are then replaced by lipids from the inner membrane. The decrease in lipids of the inner membrane leads to cell lysis (Sutterlin et al., 2016).

**Figure 3:**
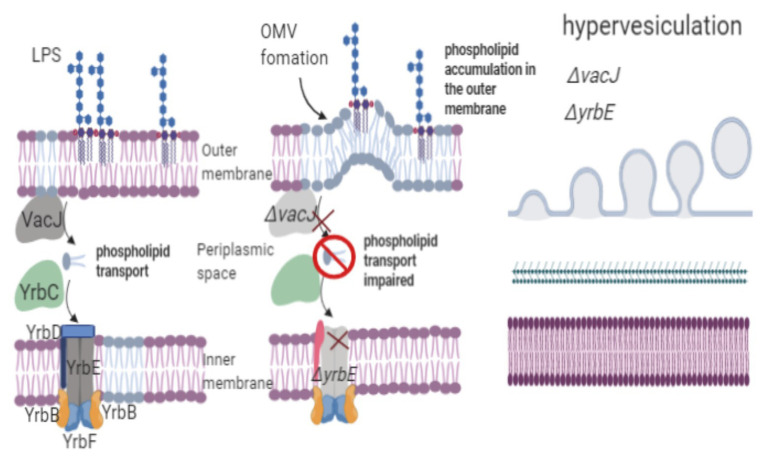
Phospholipid transport regulates OMVs biogenesis. *H. influenzae vacJ* and *yrbE* mutants show a hypervesiculation phenotype because of the accumulation of phospholipids in the outer membrane. The *vacJ* and *yrbE* genes are involved in retrograde transport, maintaining membrane asymmetry. To trigger vesicle formation in the outer membrane, the transport of phospholipids from the inner membrane to the outer membrane should be significantly faster than phospholipid transport from the outer membrane to the inner membrane. Disorganization in phospholipid transport leads to the accumulation of phospholipids in the outer membrane, provoking OMV release.

Roier et al. (2016) mutated the *vacJ* and *yrbE* genes of *Haemophilus influenzae*, which are homologous to the *mlaA* and *mlaE* genes (respectively) of *E. coli* and are involved in lipid membrane asymmetry. Vesiculation increased 1.6‐ and 2.2-fold in the *H. influenzae vacJ* and *yrbE* mutants, respectively, while vesicle production decreased in the complemented mutants. Vesicles from the *H. influenzae vacJ* and *yrbE* mutants exhibited higher levels of PE and myristic acid (C14:0), similar to the outer membrane composition in the wild-type strain; however, the PE and C14:0 fatty acid ratio increased only in vesicles produced by the mutants. Hypervesiculation in *H. influenzae vacJ* and *yrbE* was directly due to phospholipid accumulation in the outer membrane because the genes involved in retrograde transport were mutated. The *H. influenzae vacJ* and *yrbE* mutants showed the hypervesiculation phenotype, which could be caused by the regulation of the transport of phospholipids from the inner leaflet to the outer leaflet of the outer membrane. Mutation of the homologous genes *vacJ* and *yrbE*, which are associated with phospholipid transport in *V. cholerae*, *E. coli*, and *Campylobacter jejuni*, increased vesiculation, confirming the hypothesis that phospholipid asymmetry (retrograde trafficking of phospholipids) plays a role in OMV biogenesis (Roier et al., 2016; Davies et al., 2019). Homologous proteins to the phospholipid ABC transport system were found in phylogenetically distant species, such as *Pasteurella multocida*, *P. aeruginosa*, *S. typhimurium*, and *Yersinia enterocolitica*, among others. This finding suggests that the mechanism of OMV formation may be strongly linked to phospholipid transport (Roier et al., 2016). To trigger vesicle formation in the outer membrane, the transport of phospholipids from the inner membrane to the outer membrane should be significantly faster than that from the outer membrane to the inner membrane. Disorganization in phospholipid transport leads to phospholipid accumulation in the outer membrane, provoking OMV release (Figure 3).

Differences found in the composition of these phospholipids suggest that vesicles are released from the microdomains of diverse phospholipid compositions. However, the possibility of a different mechanism that can regulate phospholipids in OMVs should not be excluded. The composition of predominant phospholipids in OMVs has already been determined. For example, vesicles purified from *E. coli* ETEC exhibited high concentrations of PG, CL, and PE. By contrast, OMVs from *A. actinomycetemcomitans* contained high concentrations of CL and PE, whereas OMVs from *P. aeruginosa* only showed high concentrations of PG, and OMVs from *H. influenzae* mainly comprised PE (Kato et al., 2002). The accumulation of certain lipids, such as PG and PE, in OMVs is essential to decipher a mechanism for OMV biogenesis.

Tol-Pal helps maintain outer membrane stability through lipoprotein linkages. This system is involved in phospholipid retrograde transport and contributes to OMV biogenesis (Masilamani et al., 2018; Shrivastava and Chng, 2019). Because the *S. typhimurium tolA*, *tolQ*, and *tolR* mutants display high levels of PG and PE in the outer membrane, the presence of glycerophospholipids may promote OMV release (Masilamani et al., 2018). Because Tol-Pal proteins lack a phospholipid-binding domain, they may stabilize unidentified phospholipid transporters or act as phospholipid transporters from the inner to the outer membrane (Shrivastava and Chng, 2019).

### Bacterial Flagellin Induces OMV Release

Several studies have shown an association between the bacterial flagellum and OMVs. The flagellum comprises a basal body, a flexible linker known as the hook, and a filament that drives the bacterium’s movement. The filament of the bacterial flagellum comprises the flagellin protein (Chen et al., 2011). Using a density gradient protocol, OMVs from *E. coli* were purified, and flagellin (FliC) was identified using proteomics (Lee et al., 2007). Manabe et al. analyzed vesicles from *E. coli* W3110 and the *E. coli ΔfliC* mutant derived from the W3110 strain. OMVs were obtained using a density gradient protocol. Both flagellin and OMVs from the wild-type strain were found in the same fraction collected from the density gradient. Moreover, flagellin was detected in the lumen of fractioned vesicles from the wild-type strain, and only in the mutant did OMV production decrease (Manabe et al., 2013). In another study, researchers tried to correlate the presence of flagella and OMV production in *Vibrio fischeri*; in this bacterium, the flagellum shaft is encased by a membranous sheath (Millikan and Ruby, 2004; Aschtgen et al., 2016). In this study, vesicle production was determined in the *V. fischeri motB1* mutant (MotB1 is a sodium pump and the main motor protein for the flagellum machinery), *V. fischeri* nonflagellated *flrA* mutant (*flrA* codes for a transcriptional activator), *V. fischeri* hyperflagellated swarmer strain (expresses 3‐ to 4-fold more flagella), and *V. fischeri* wild-type strain. However, the number of vesicles decreased in the *V. fischeri flrA* mutant that lacks flagella. Vesicle production increased in the hyperflagellated strain. The addition of phenamil (a sodium-pump blocking reagent) to the *V. fischeri* hyperflagellated strain decreased OMV production, suggesting that flagellum rotation improves vesicle release (Aschtgen et al., 2016). To demonstrate the association of flagella with OMV production, the following organisms were analyzed: *V. fischeri* and *V. parahaemolyticus* (which expresses several sheathed flagella), *V. cholerae* (which contains unique polar sheathed flagella), *E. coli* (which expresses an unsheathed flagellum), and the *V. fischeri* nonflagellated *flrA* mutant. *V. fischeri* and *V. parahaemolyticus* released more vesicles than *V. cholerae* and *E. coli*, while the *V. cholerae* nonflagellated mutant exhibited decreased OMV production (Aschtgen et al., 2016). They concluded that species expressing flagella such as *E. coli* and *Vibrio* released more vesicles. Clearly, unsheathed and sheathed flagella perturb the outer membrane and promote OMV release. To better ascertain the relationship between flagella and OMV production, more flagellated strains along with their corresponding nonflagellated mutants and hyperflagellated strains should be studied. It is important to explore whether flagellum movement only promotes the release of vesicles from the surrounding outer membrane area or promotes vesiculation from the whole cell body.

### *Pseudomonas* Quinolone Signaling and Its Role in OMV Biogenesis

Bacterial species such as *P. aeruginosa* regulate the secretion of virulence factors by sensing bacterial density through the quorum-sensing mechanism (Strateva and Mitov, 2011). *P. aeruginosa* produces a quinolone called the *Pseudomonas* quinolone signal (PQS). To regulate the expression of some genes, PQS binds to the PqsR receptor (Lin et al., 2018). The synthesis of PQS is regulated by *pqsABCDE* along with other genes, including *phnAB* and *pqsH*. PqsA triggers PQS synthesis. PqsB, PqsC, and PqsD then produce 2-heptyl-4-quinolone (HHQ) or quinolone and other precursors. Finally, PqsH catalyzes the conversion of HHQ into PQS. The *P. aeruginosa pqsH* mutant synthesizes quinolones but not PQS (Lin et al., 2018).

The quorum-sensing system in *P. aeruginosa* comprises quinolone molecules, including 3-oxo-dodecanoyl homoserine lactone (3OC12-HSL), butyryl homoserine lactone (C4-HSL), and 2-heptyl-3-hydroxy-4-quinolone (PQS; Déziel et al., 2004). OMVs from *P. aeruginosa* contain higher PQS (86%) than 12-HSL and C4-HSL, which were measured using thin-layer chromatography (TLC) and mass spectrometry (Mashburn-Warren and Whiteley, 2005). Additionally, the *P. aeruginosa pqsA* mutant (displaying defective quinolone synthesis) showed a 10-fold decrease in OMV production. Vesicle production was restored after treating mutants with a synthetic quinolone. Thus, PQS may also be involved in OMV release. However, when the *P. aeruginosa mvfR* mutant (which lacks the PQS *mvfR* regulator) was treated with exogenous PQS, it yielded the same number of vesicles as that of the wild-type strain. This finding suggests physical interactions among PQS and different undetermined molecules found in outer membrane sites where it begins to bulge (Mashburn-Warren and Whiteley, 2005). Additionally, the interaction of LPS with either PQS or the alkyl chain and hydroxyl group (-OH) of PQS induced a curvature in the outer membrane necessary to start vesicle formation. Hence, a synthetic compound similar to PQS (which lacks the alkyl chain and hydroxyl group) does not interact with LPS, while the natural PQS molecule interacts with LPS (Mashburn-Warren et al., 2008). Moreover, PQS could impair the interaction among divalent cations (Mg^+2^ and Ca^+2^) and phosphate at the 4-position of lipid A, affecting the motility of lipid A and destabilizing the outer membrane, improving outer membrane protrusion and vesicle release (Bredenbruch et al., 2006; Mashburn-Warren et al., 2008).

In 2012, Schertzer and Whiteley proposed a novel bilayer-coupled model to explain OMV biogenesis, which they theorized could be mediated by PQS in *P. aeruginosa* (Schertzer and Whiteley, 2012). This model requires the presence of an amphiphilic molecule that, due to specific electric interactions, would concentrate in the outer leaflet of the membrane, leading to the expansion of the outer leaflet while promoting membrane curvature (Sheetz and Singer, 1974). To study the effect of small molecules on the cell membrane, a solution prepared with a low concentration of PQS was added to red blood cells. The erythrocytes showed crenation and membrane protrusions, an effect similar to that elicited during vesicle production (Schertzer and Whiteley, 2012). The addition of chlorpromazine, which induces cup formation in red blood cells, prevented the formation of protrusions in the membrane. Such evidence indicates that PQS may contribute to OMV release, in accordance with the bilayer-coupled model proposed by Schertzer and Whiteley (2012).

*P. aeruginosa* PA01 strain was previously shown to produce a higher concentration of PQS in the inner membrane and released fewer OMVs. By contrast, PQS production in *P. aeruginosa* PA14 was extracellular, and a higher OMV production was observed. These results suggest that a relevant difference between the strains could be defective PQS export (Florez et al., 2017). Temporal analyses showed PQS accumulation in the inner membrane of the *P. aeruginosa* PA01 strain due to PQS saturation. This saturation was observed after culturing the strain for 10 h in Luria-Bertani medium (Florez et al., 2017). When the PA01 strain was cultured using brain infusion broth, vesicle production increased along with PQS export compared with that obtained in Luria-Bertani medium. These results were similar to those reported in clinical strains of *P. aeruginosa* isolated from patients with cystic fibrosis. These strains showed an increase in OMV production and PQS export in brain infusion broth culture (Florez et al., 2017). Future research is required to analyze PQS transport in Gram-negative clinical strains and define possible PQS-associated mechanisms in OMV biogenesis.

In 2018, Horspool and Schertzer treated different strains of gammaproteobacteria, such as *E. coli*, *Klebsiella pneumoniae*, and *Proteus mirabilis*, with exogenous PQS. All the strains increased vesicle production up to 3.5-fold. This effect was not observed in alphaproteobacteria such as *Agrobacterium tumefaciens* and *Caulobacter crescentus*. Importantly, the authors observed an increase in OMV production when using the *P. aeruginosa* strain that was stimulated with a concentrated supernatant from cultures of *E. coli* and *K. pneumoniae* (Horspool and Schertzer, 2018). Molecules present in the supernatants induced OMV production in *P. aeruginosa*, but these molecules have yet to be identified. Although Mashburn-Warren and Whiteley attributed OMV production in *P. aeruginosa* exclusively to PQS, they also observed a minimal concentration of acyl-homoserine lactone packaged in OMVs. Therefore, exogenous acyl-homoserine lactone from *E. coli* and *K. pneumoniae* induce membrane curvature, leading to OMV release in *P. aeruginosa* (Mashburn-Warren and Warren, 2005; Schertzer and Whiteley, 2012).

## Concluding Remarks

Several works have focused on explaining the different possible OMV biogenesis mechanisms. Lipoproteins, outer membrane proteins, LPS, and flagellin have been proposed as the key pieces in different mechanisms of OMV biogenesis.

Lipoproteins and outer membrane proteins involved in linkages within the bacterial envelopes regulate membrane protrusion and subsequent OMV release. Further investigation is needed and should focus on the search for lipoproteins and outer membrane proteins homologous to those reviewed here involved in OMV biogenesis. The interaction with the peptidoglycan and/or outer membrane and null mutants were then analyzed to determine their role in vesicle formation. It is possible that more than one molecule, such as LPS, outer membrane proteins, and phospholipids, could contribute to vesicle formation. Although LPS differs considerably among bacterial species, it has also been associated with OMV biogenesis. LPS charge and architecture are involved in OMV formation and protein composition. In addition, it should be explored which LPS component (O-antigen, core, and/or lipid A) plays the major role in vesiculation. However, retrograde phospholipid transport is involved in OMV release; phospholipid accumulation increases outer membrane protrusion and subsequent vesicle release. Other molecules, including PQS or flagellin, could be involved in vesicle formation. Although the secretion of PQS molecules or presence of flagella in the cell body is restricted to a few bacterial species, their role in vesiculation must be explored in more detail.

## Author Contributions

All authors listed have made a substantial, direct and intellectual contribution to the work, and approved it for publication.

### Conflict of Interest

The authors declare that the research was conducted in the absence of any commercial or financial relationships that could be construed as a potential conflict of interest.
